# The individual and sequential effect of focused attention and open monitoring meditation on mindfulness skills

**DOI:** 10.1371/journal.pone.0322537

**Published:** 2025-05-07

**Authors:** Haruyuki Ishikawa, Toshizumi Muta, Tetsuri Abe, Nozomi Imajo, Fusako Koshikawa

**Affiliations:** Faculty of Letters, Arts and Sciences, Waseda University, Tokyo, Japan; Bangladesh Open University, BANGLADESH

## Abstract

Mindfulness meditation has two components: focused attention (FA) meditation and open-monitoring (OM) meditation. Based on traditional Buddhist principles, it is recommended that FA meditation be practiced prior to OM meditation. However, the influence of the order in which these meditations are practiced on the efficacy of interventions has not yet been empirically investigated. This study examined the effects of the order of FA and OM meditation on the level and process of acquiring mindfulness skills. Thirty-three Japanese undergraduate and graduate students who were meditation novices completed the intervention. Participants were assigned to three groups: the group practicing 4-week FA meditation prior to 4-week OM meditation (FA-OM group), the group practicing meditation in reverse order (OM-FA group), and the wait-list control group. Each meditation training session consisted of one 1-hour group session per week and a 15-min daily homework. All participants were assessed for trait mindfulness once a week. The results showed that the FA-OM group scored higher than the control group for most mindfulness skills. In addition, awareness, describing, acceptance, and observing skills increased earlier in the FA-OM group than in the OM-FA group. However, the skill of staying aware at the present moment increased earlier in the OM-FA group than in the FA-OM group. These results suggest that the order of practicing the two meditations primarily influences the process of acquiring mindfulness skills rather than the level of skills, and that it is reasonable to practice FA meditation prior to OM meditation to cultivate mindfulness skills for novices.

## Introduction

Contemporary research has extensively investigated the beneficial effects of meditation on health. Currently, one form of meditation that has been extensively applied as a psychological intervention is mindfulness, defined as “paying attention in a particular way, on purpose, in the present moment, and nonjudgmentally” [[1], p. 4]. Research has shown that mindfulness meditation is an effective intervention for improving the quality of life of healthy individuals [2] and alleviating symptoms associated with psychological distress, major depression, anxiety disorder, addictive disorder, and pain. [3,4].

Mindfulness meditation comprises two types of meditation: focused attention (FA) meditation, which requires concentrating and sustaining attention on an intended object such as one’s breath, and open monitoring (OM) meditation, which requires monitoring anything occurring in one’s experience without focusing on a particular object [5]. These correspond to samatha (śamatha) and vipassana (vipaśyanā) in Buddhist philosophy, respectively.

Studies examining the effects of FA and OM meditations suggest that they are associated with different cognitive processes [6–9]. It is reported that an 8-week FA meditation training particularly heightens self-reported attention control skills, whereas OM meditation training heightens self-reported emotional non-reactivity skills [10]. Another study showed that the ability to overcome habitual behavior was facilitated in both FA and OM meditation training, but more in the latter [11]. An examination of the brain activity of expertized meditators during meditation shows that OM rather than FA meditation is associated with decreased intentional attention focus and increased non-judgmental and non-reactive attitude [6]. The meta-analysis of neuroimaging studies showed that FA meditation is associated with increased top-down attention regulation and decreased mind wandering; meanwhile, OM meditation is associated with increased monitoring of internal/external information and decreased selective filtering of sensory signals [12]. Thus, FA meditation would strongly foster voluntary attention regulation and OM meditation would cultivate the attitude of non-judgment and acceptance.

The training of these two meditations would also lead to different emotion regulation strategies. It was reported that although both meditations increased acceptance, FA meditation increased the frequency of reappraisal and calming strategies compared to OM meditation [13]. An electroencephalogram (EEG) study showed that of the two only FA meditation training decreased frontal alpha asymmetry, which reflects emotional reactivity to emotional stimuli [14].

Even brief FA and OM meditations can induce differential physiological responses. Ooishi et al. [15] showed that a 30-min FA meditation increased cardiac parasympathetic modulation, whereas OM meditation increased cardiac sympathetic modulation and decreased salivary cortisol levels in novices. Furthermore, it was reported that brief OM meditation initially evoked a higher level of emotional response to negative stimuli than the distraction strategy, but the response gradually decreased with repetitive trials [16]. As mindfulness-based interventions are a mixture of these different components, their effects are confounded by the outcome. The need to reveal the role of each type of meditation separately was suggested from a clinical perspective [17]. In particular, it would be beneficial to examine how the FA and OM components play a role in the acquisition of mindfulness.

Although the individual effects of FA and OM meditation have been clarified in recent studies, their combined effects remain unclear. In particular, we focused on how the order of practicing meditation influenced the acquisition of mindfulness skills in meditation novices. According to Buddhist principles, concentration is the basic component of vipassana meditation [18]. Therefore, practicing FA meditation followed by OM meditation is recommended [5]. Mindfulness-based stress reduction (MBSR) also addresses FA before OM skills [19]. It was reported that FA meditation experts showed higher executive function, whereas OM meditation experts showed higher attentional orientation and executive function compared to non-meditators [20]. This finding supports the view that OM meditation is based on skills cultivated through FA meditation. Another study showed that a single session of FA meditation increased attentional function in the sample with lower mindfulness skill, suggesting that FA meditation is more suitable for novices than OM meditation [21].

However, to the best of our knowledge, no empirical studies have examined the effects of the order of the two meditations. The only study focusing on the role of FA and OM components in mindfulness intervention examined the effect of an 8-week program consisting of a 4-week FA meditation-based training program and a subsequent 4-week OM meditation-based training program on trait mindfulness and emotion regulation [22]. They found that mindfulness, depression and anxiety symptoms, and executive function improved during FA meditation training and were maintained during OM meditation training in novices. Nevertheless, the study did not examine the effect of meditation training in the reverse order. Additionally, the meditation program used in this study included several techniques and was not separated into pure FA and OM meditation periods. Thus, it is difficult to conclude whether practicing FA meditation before OM meditation is preferable for novices.

The present study aimed to examine how differences in the order of FA and OM meditation training in an 8-week training influences the acquisition of mindfulness skills in novices. We directly compared two meditation groups: a group practicing FA meditation before OM meditation and a group practicing OM meditation before FA meditation. According to Buddhist tradition, it was hypothesized that the former group would show higher levels or earlier improvement in mindfulness skills than the latter group. Furthermore, trait mindfulness includes several components, such as awareness, describing, and non-reactivity [23]. It has been suggested that FA and OM meditation increase different aspects of mindfulness skills [10]. Thus, we sequentially measured each component of mindfulness during meditation training to compare the process of acquiring mindfulness skills between the two groups. We hypothesized that the aspect of mindfulness skills concerning top-down attention regulation (i.e., awareness, observing and describing one’s sensations or feelings, and focusing on present moment) particularly improve earlier and higher in the group practicing FA meditation first than the group practicing OM meditation first and the non-meditation group.

## Methods

### Participants

We first conducted a priori power analysis using the software “G*Power version 3.1.9.7” [24] to determine the sample size. We assumed a small to medium effect size (partial eta square = .02 to.06). Type 1 error rate and statistical power (1 - *β*) were set as 0.05 and 0.95, respectively. The number of groups was three, and the number of repeated measurements was nine. The results showed that 9–28 participants were required in each group.

The study design was a non-randomized controlled trial. Participants were recruited through advertisements placed on a Japanese university campus as well as a website for part-time jobs at the university. The exclusion criteria were those: (a) who have previously participated in any mindfulness or other meditation program, and (b) who declared to have a diagnosis of developmental disorder or mental illness. We set up four one-hour meditation classes (two classes for one meditation group and two classes for another meditation group) per week. Participants were asked which class they could attend and whether they consented to the measurement of their brain activity in addition to answering the questionnaire. Based on their available schedule, participants were assigned to three groups: the group practicing FA meditation prior to OM meditation (FA-OM group), the group practicing the two meditations in reverse order (OM-FA group), or the wait-list control group. The rate of participants who agreed with the measurement of their brain activity was balanced in each group. Thirty-three undergraduate and graduate students participated in the program (11 males and 22 females). Six of whom dropped out (two of them were due to scheduling conflict, and the reason could not be identified for the rest). In total, 27 participants (9 males and 18 females; age = 19.75 ± 1.45 years) completed the entire study. Each group comprised nine participants. All participants were Asian and could understand and speak Japanese. One participant (in the control group) was a graduate student, while the rest were undergraduates. Two participants in the OM-FA group and one in the control group previously had the opportunity to experience Zen meditation and yoga. Sample characteristics were presented in Table 1. No other demographic details were collected.

**Table 1:** Sample characteristics.

	FA-OM	OM-FA	Control	Total
**Female, n(%)**	5(55.6%)	6(66.7%)	6(66.7%)	17(63.0%)
**Race, n(%)**				
Asian	9(100%)	9(100%)	9(100%)	27(100%)
Others	0(0.0%)	0(0.0%)	0(0.0%)	0(0.0%)
**Age, mean(SD)**	19.6(1.2)	19.2(1.1)	20.3(1.8)	19.7(1.5)
**Grade, n(%)**				
Undergraduate	9(100%)	9(100%)	8(88.9%)	26(96.3%)
Graduate	0(0.0%)	0(0.0%)	1(11.1%)	1(3.7%)
**Past Experience, n(%)**				
Meditation	0(0.0%)	1(11.1%)	1(11.1%)	2(7.4%)
Yoga	0(0.0%)	1(11.1%)	0(0.0%)	1(3.7%)
No	9(100%)	7(77.8%)	8(88.9%)	24(88.9%)

### Meditation interventions

The meditation instructions used in this study were made by one of the authors who has over 35 years of meditation experience; they were also based on the procedures in the literature on counting breaths meditation used in Zen Buddhism [25] for FA meditation and on Vipassana meditation [26] for OM meditation. The participants were provided with face-to-face and audio-based instructions on how to meditate. In the face-to-face session, FA and OM meditations were instructed by three experimenters: one had completed several mindfulness teacher courses and practiced meditation for over 35 years, another had completed the 8-week Mindfulness Self-Compassion course, and the other had completed 21 hours of meditation sessions under the supervision of certified mindfulness trainers. Two of the three experimenters attended each session. Participants also received 15-min of audio instruction and a paper manual for each meditation for use in daily homework.

### FA meditation

FA meditation in the present study was based on breath-counting meditation. Participants were instructed to take a breath slowly and count their breaths from one to ten in their minds, and repeat. When they were aware of being distracted, they were encouraged to redirect their attention to their breath and start counting from one again.

### OM meditation

In the present study, OM meditation emphasized observing one’s internal/external stimuli and responses to them, with a live description of what happens sub vocally. The participants were instructed to observe and describe their breaths, sensations, moods, and thoughts moment by moment. The S1 Appendix contains additional detailed information on the instructions for these meditations.

### Measure

The Six-Factor Mindfulness Scale (SFMS) [27] was used to assess trait mindfulness. This Japanese scale was based on the Five Facet Mindfulness Questionnaire [28], which is the most widely used questionnaire for the measurement of trait mindfulness, and was particularly developed to be applicable to meditation novices. It is comprised of six subscales: *Nonduality,* which assesses the attitude to sympathize with oneself and others to the same degree (i.e., I can sympathize with myself and others equally), *Describing,* which assesses the ability to verbalize and label one’s physical sensations, feelings, and thoughts (i.e., I can describe what I feel accurately in words), A*cceptance and Nonreactivity,* which assesses the attitude to accept internal/external events as it is without responding automatically or judging (i.e., I let go of my thoughts and feelings without devoting myself to them), *Objective Observing,* which assesses the attitude to objectify and observe one’s experiences (i.e., I can observe my feelings objectively), *Awareness,* which assesses the attitude to direct attention to one’s internal events and to act with awareness (i.e., I am aware of my feelings moment by moment), and *Being in The Moment,* which assesses the attitude to intentionally focus on experiences in the present moment rather than the past or future (i.e., I think about what I do more frequently than past events). The subscales included three, three, nine, three, seven, and six items, respectively. All items were rated on a 5-point Likert scale (1 = “not applicable at all” to 5 = “very applicable”). This questionnaire is shown to have adequate reliability (Cronbach’s α = .86 (total score) and.70 to.83 (each subscale)) and validity.

### Design and procedure

**Fig 1:**
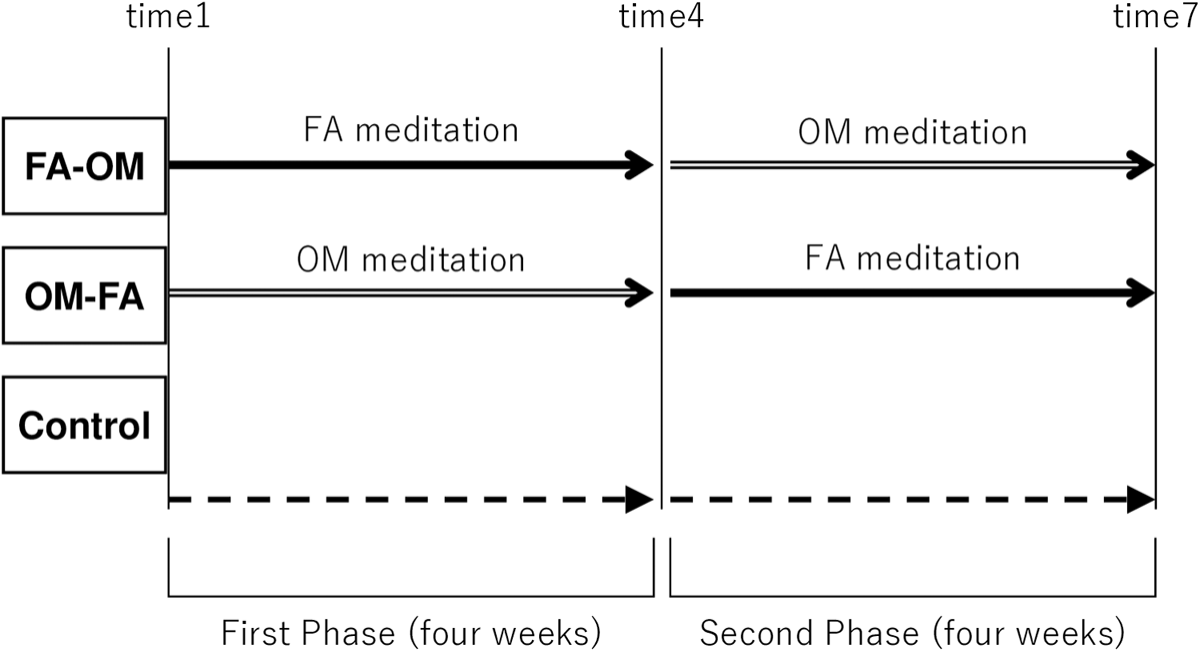
Procedure of the study.

Fig 1 illustrates the study procedure. Participants in the FA-OM group practiced FA meditation for four weeks (first phase), followed by OM meditation for four weeks (second phase). Alternatively, participants in the OM-FA group practiced the 4-week OM meditation followed by the 4-week FA meditation. Each meditation training session consisted of one 1-hour group session per week and a 15-min daily homework practice. The first session (time 1) included obtaining written informed consent, an explanation of the outline of the present study, and the first meditation practice following the baseline measurement of the SFMS. The measurement took about 5–10 minutes. The participants were asked to write about what they felt or thought during their daily homework practice and send it via email or a messaging app every day to ensure that they continuously engage in meditation. The following sessions (time 2–9) comprised of the measurement of the SFMS, meditation practice, and sharing in-session and homework practices. Participants in the control group provided written informed consent and completed the SFMS once per week without any intervention. The study took place between 20 May and 31 July 2019.

Participants in the two meditation groups received 10,000 JPY, and those in the control group received 5,000 JPY, except for two participants who refused to accept the payment. We also measured the brain activity of some participants using near-infrared spectroscopy while they engaged in cognitive tasks (omitted in this paper). These participants were paid an additional 10,000 JPY. The payment was based on the hourly rate for the time required to perform the procedure, determined in accordance with regulations of the university.

This study was approved by the Ethics Review Committee on Research with Human Subjects of [blinded for review] and conducted according to the Declaration of Helsinki.

### Analyses

To examine the differences in trait mindfulness between the groups, a series of two-way ANOVAs (group × time) was performed for each subscale of the SFMS. This analysis was conducted using the software “HAD” [29].

Next, we treated each data as a single-case data because the outcomes were repeatedly measured, and the sample size was relatively small. Multilevel modeling was conducted to estimate the change in SFMS scores within the first and second phases. It is commonly used to integrate the results of multiple single cases [30]. We conducted two-level regression analyses, which included repeated measurements (Level 1) nested within subjects (Level 2), for the two meditation groups separately. The intercept, time, phase, and their interactions were entered into the models as fixed and random effects (case-specific deviation). The time variable was centered on setting the first observation of the second meditation phase (time 6) to zero. The time coefficients indicate an increase in scores for each additional time point within the first phase. For the phase variables, the first phase was coded as zero and the second phase as one. The coefficients of the phase indicated an increase in scores in the first week of the second phase. The time × phase coefficients reflect the change in trends between the first and second phases. This analysis was conducted using the online tool “MultiSCED (http://34.251.13.245/MultiSCED/)” [31].

Finally, we calculated *Tau-U*, which is an index of non-overlap between two periods (i.e., baseline and intervention) controlling for trends in the baseline period [32]. In the present study, the first and second phases were considered the baseline and intervention periods, respectively. Thus, the index shows the effect size of the extent to which the scores in the second meditation phase were superior to those in the first meditation phase. *Tau-U* less than 0.20, 0.20 to 0.60, 0.60 to 0.80, and above 0.80 are considered as a small, moderate, large, and very large change, respectively [33]. Averaged *Tau-U* and *p*-value in each group were calculated using the online tool “Tau-U Calculator (http://singlecaseresearch.org/calculators/tau-u)” [34].

## Results

### Two-way mixed ANOVA and ANCOVA

**Fig 2:**
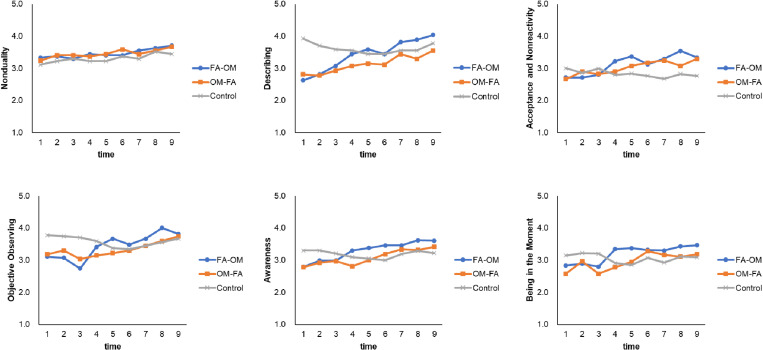
The scores of each subscale of SFMS during the eight weeks.

The SFMS scores at each time point are shown in Fig 2 and S1 Table. First, a series of two-way (group × time) mixed ANOVAs were conducted for each SFMS subscale (Table 2). The interactions for all subscales except *Nonduality* were significant. Although there were significant simple main effects of the group for *Describing* at time 1 and 2 and for *Observing* at time 3, no difference between the groups was found at time 9. The simple main effects of time were significant in all groups for *Awareness* and were significant only in the FA-OM and OM-FA groups for *Describing*, *Being in The Moment*, *Observing*, and *Acceptance*. We then compared the scores between time 1 and other time points to detect the first significant increase in trait mindfulness in each group (Table 3). The *p*-values were adjusted by the Bonferroni method (*α* = 0.05/8). *Awareness*, *Describing*, and *Acceptance* increased at earlier time points in the FA-OM group (time 5, 4, and 5, respectively) than in the OM-FA group (time 7, 9, and 6, respectively). *Observing* increased at time 8 in the FA-OM group, whereas no change was found in the OM-FA group. On the other hand, for *Being in The Moment*, OM-FA group showed the increase at time 6, whereas the FA-OM group showed an increase at time 9. No significant changes from baseline were observed in the control group.

**Table 2:** Two-way ANOVAs for each subscale of SFMS.

	Group	Time		Group × Time	
Nonduality	*F*(2, 24) = 0.22	*F*(8, 192) = 2.46	^†^	*F*(16, 192) = 0.22	
Describing	*F*(2, 24) = 1.27	*F*(8, 192) = 7.79	^***^	*F*(16, 192) = 4.00	^***^
Acceptance and Nonreactivity	*F*(2, 24) = 0.40	*F*(8, 192) = 4.49	^***^	*F*(16, 192) = 3.99	^***^
Objective Observing	*F*(2, 24) = 0.33	*F*(8, 192) = 4.81	^***^	*F*(16, 192) = 3.25	^***^
Awareness	*F*(2, 24) = 0.32	*F*(8, 192) = 7.46	^***^	*F*(16, 192) = 3.36	^***^
Being in the Moment	*F*(2, 24) = 0.26	*F*(8, 192) = 4.23	^**^	*F*(16, 192) = 2.95	^**^

† *p* < .10,

*p < .05,

**p < .01,

***p < .001.

**Table 3:** Multiple comparisons for each subscale of SFMS.

	FA-OM	OM-FA	Control
Nonduality	–	–	–
Describing	t1 > t4, t5, t6, t7, t8, t9	t1 > t9	–
Acceptance and Nonreactivity	t1 > t5, t7, t8, t9	t1 > t6, t7, t9	–
Objective Observing	t1 > t8	–	–
Awareness	t1 > t5, t6, t7, t8, t9	t1 > t7	–
Being in the Moment	t1 > t9	t1 > t6, t9	–

The scores at time 1 (t1) were compared to those at other time points. All differences between pairs listed in the table were significant (*ps* < .05).

To compare the scores between groups, controlling for the difference at baseline, we additionally conducted a series of ANCOVAs (including group and time (time 2–9) as independent values and baseline scores as covariates) and post-hoc analyses for each subscale of the SFMS. Significant interactions were found in all subscales except for *Nonduality*. *Describing* was higher in the FA-OM group than in the control group at all time points from time 5–9 and compared to the OM-FA group at time 8 (*ps* < .05). *Acceptance* was higher in FA-OM group than control group at all time points from time 4 to time 9 (*ps* < .05). The OM-FA group also had higher scores than the control group at time 6, 7, and 9 (*ps* < .05). *Observing* was higher in the FA-OM group than in the control group at time 8 (*p* = .02). *Awareness* was higher in the FA-OM group than in the control group at all time points from time 5–9 (*ps* < .05). *Being in The Moment* was higher in the FA-OM group than in the control group at time 4, 5, and 7 (*ps *< .05). The OM-FA group was also higher than the control group at time 6 and 7 (*ps *< .05).

### Multilevel modeling

Second, multilevel modeling was conducted in FA-OM and OM-FA groups. Table 4 presents the results of the analyses. For *Awareness*, the main effect of the time was marginally significant in the FA-OM group, indicating that the average score increased by 0.15 point per time point within the first FA meditation phase, up until 3.54 point at the start of the second OM meditation phase. However, the second OM meditation showed no immediate effect (time 6) or any interaction with the first FA meditation. Similarly, the FA-OM group showed a significant increase in *Describing* and a marginally significant increase in *Being in The Moment* within the FA meditation training. Regarding *Acceptance*, the average score significantly increased within the FA meditation training in the FA-OM group and increased at a marginally significant level within the OM meditation training in the OM-FA group. There was no immediate effect of the second meditation training or interaction between the first and second meditations on any SFMS subscales.

**Table 4:** Multilevel modeling for each subscale of SFMS in FA-OM and OM-FA group.

FA-OM												
	Nonduality		Describing		Acceptance and Nonreactivity		Objective Observing		Awareness		Being in the Moment	
*Fixed Effects*	Estimates (SE)		Estimates (SE)		Estimates (SE)		Estimates (SE)		Estimates (SE)		Estimates (SE)	
Intercept	3.44 (0.17)	^***^	3.86 (0.18)	^***^	3.51 (0.25)	^***^	3.63 (0.19)	^***^	3.54 (0.23)	^***^	3.51 (0.28)	^***^
time	0.02 (0.05)		0.25 (0.07)	^*^	0.18 (0.07)	^*^	0.14 (0.08)		0.15 (0.07)	^†^	0.15 (0.07)	^†^
phase	-0.01 (0.18)		-0.30 (0.20)		-0.32 (0.21)		-0.09 (0.21)		-0.09 (0.19)		-0.22 (0.21)	
time × phase	0.07 (0.07)		-0.10 (0.09)		-0.09 (0.08)		-0.01 (0.10)		-0.09 (0.07)		-0.10 (0.07)	
***Random Effects***	*SD*		*SD*		*SD*		*SD*		*SD*		*SD*	
Intercept	0.38		0.39		0.67		0.36		0.64		0.76	
time	0.13		0.19		0.19		0.21		0.19		0.18	
phase	0.29		0.29		0.50		0.30		0.44		0.46	
time × phase	0.10		0.19		0.18		0.19		0.15		0.11	
Residual	0.33		0.37		0.29		0.41		0.26		0.32	
OM-FA												
	Nonduality		Describing		Acceptance and Nonreactivity		Objective Observing		Awareness		Being in the Moment	
*Fixed Effects*	Estimates (SE)		Estimates (SE)		Estimates (SE)		Estimates (SE)		Estimates (SE)		Estimates (SE)	
Intercept	3.49 (0.20)	^***^	3.24 (0.35)	^***^	3.12 (0.30)	^***^	3.16 (0.35)	^***^	3.00 (0.28)	^***^	2.93 (0.30)	^***^
time	0.04 (0.05)		0.10 (0.06)		0.08 (0.04)	^†^	-0.01 (0.05)		0.03 (0.03)		0.06 (0.04)	
phase	0.03 (0.14)		-0.06 (0.26)		0.05 (0.17)		0.14 (0.18)		0.22 (0.14)		0.30 (0.22)	
time × phase	0.00 (0.06)		0.02 (0.07)		-0.06 (0.08)		0.16 (0.11)		0.03 (0.07)		-0.09 (0.07)	
***Random Effects***	*SD*		*SD*		*SD*		*SD*		*SD*		*SD*	
Intercept	0.56		0.99		0.86		1.01		0.8		0.81	
time	0.14		0.14		0.09		0.13		0.06		0.07	
phase	0.26		0.65		0.36		0.37		0.26		0.46	
time × phase	0.15		0.12		0.20		0.31		0.16		0.09	
Residual	0.23		0.33		0.28		0.29		0.16		0.35	

† *p* < .10,

*p < .05,

***p < .001

### Tau-U analysis

Third, *Tau-U* between the first and second phases were calculated. In accordance with the recommendation in Vannest and Ninci [33], the first phase trends over 0.10 were adjusted. The combined *Tau-U* averages were calculated for the FA-OM, OM-FA, and control groups. Table 5 presents the summary of the results. For *Nonduality*, only the second OM meditation showed a moderate increase at a marginally significant level in the FA-OM group. In contrast, only the second FA meditation showed a moderate increase of *Being in The Moment* in OM-FA group. For *Awareness*, *Describing*, *Observing*, and *Acceptance*, the second meditation phase showed moderate increases in both the FA-OM and OM-FA groups. The control group showed no significant increase but a moderate decrease in *Acceptance* in the second phase.

**Table 5:** The Averaged Tau-U comparing the SFMS scores in second phase with the first phase.

	FA-OM			OM-FA			Control		
	*Tau-U*		90%CI	*Tau-U*		90%CI	*Tau-U*		90%CI
Nonduality	0.23	^†^	0.01–0.46	0.22		-0.01–0.44	0.22		-0.01–0.44
Describing	0.38	^**^	0.15–0.60	0.31	^*^	0.09–0.54	0.03		-0.20–0.25
Acceptance and Nonreactivity	0.26	^*^	0.04–0.49	0.41	^**^	0.19–0.64	-0.29	^*^	-0.52 – -0.07
Objective Observing	0.34	^*^	0.12–0.56	0.41	^**^	0.19–0.64	-0.13		-0.35–0.10
Awareness	0.39	^**^	0.17–0.62	0.51	^***^	0.29–0.74	0.10		-0.12–0.32
Being in the Moment	0.21		-0.02–0.43	0.57	^***^	0.34–0.79	0.18		-0.04–0.41

† *p* < .10,

*p < .05,

**p < .01,

***p < .001

## Discussion

This study examined how the order of FA and OM meditation training influenced novices’ acquisition of mindfulness skills. The levels and patterns of changes in trait mindfulness were compared between the FA-OM group (4-week FA meditation followed by 4-week OM meditation), the OM-FA group (4-week OM meditation followed by 4-week FA meditation), and the wait-list control group. Unexpectedly, we found that the control group showed a significantly higher *Describing* score than the two meditation groups at baseline; there was no difference between the groups at the end of the training (time 9). However, the FA-OM group showed higher scores for most aspects of mindfulness skills than the control group when controlling for the baseline score. In addition, we found the possibility that the order of the two different meditation training influenced the process of acquiring mindfulness skills, partly supporting our hypothesis. Notably, however, we could not confirm if the differences between groups were not due to inequality of any demographic variables since this was a non-randomized study.

### The individual and sequential effects of two meditations

Novices who practiced FA meditation prior to OM meditation showed a significant increase in *Acceptance*, *Awareness*, and *Describing* at earlier time points compared to novices who practiced the two types of meditation in reverse order. In the FA-OM group, these skills increased within the former 4-week FA meditation training. There were also positive slope coefficients for *Acceptance* and *Describing* scores (and for *Awareness* at a marginally significant level) within the FA meditation phase of the FA-OM group. FA meditation is associated with not only increased selective attention but also decreased emotional reactivity [5]. As FA meditation requires concentration on a specific internal target (i.e., breath), individuals who trained in this form of mediation first could improve their ability to sustain attention to one’s feelings or thoughts without being immersed in it. However, the results are inconsistent with those of a recent study [10], which reported that an 8-week OM meditation is superior to FA meditation in heightening non-reactive describing and awareness skills. This may be partly due to the differences in the period of meditation. In the case of a brief intervention within four weeks, FA meditation would be a more appropriate training for cultivating skills for being conscious of and accepting (and possibly keeping awareness of) one’s experiences than OM meditation.

Furthermore, the FA-OM group showed a higher *Describing* score than the OM-FA group at time 8, controlling for the baseline score. This would suggest that the ability to concentrate on a specific target, cultivated through FA meditation, facilitates more precise monitoring of one’s experiences when connected with OM meditation training. Although we instructed the participants to describe their feelings and thoughts verbally during OM meditation training, this might be difficult without selective attention training.

*Observing* increased only in the FA-OM group. Although the trend for the *Observing* score was not significant in either the FA-OM or OM-FA group, the slope coefficient within the FA meditation phase in the FA-OM group was positive, whereas that within the OM meditation phase in the OM-FA group was negative. These results would suggest that the ability to objectively observe one’s feelings and thoughts is primarily based on deliberate attention focusing and distractor inhibition. An 8-week OM meditation program was found to be inferior to FA meditation in cultivating emotion regulation skills [13] and did not change neural activity concerning emotional reactivity and emotion regulation [14]. Thus, OM meditation training before FA meditation might hinder novices from acquiring the skills to observe internal and external events from an emotionally neutral perspective.

In the OM-FA group, the slopes of trends of *Being in The Moment* within both phases were not significant. However, the effect size of the increase from the first to the second phase in the OM-FA group was larger than that in the FA-OM group. This means that participants in the OM-FA group showed a sudden increase in this skill when they began to practice the second (FA) meditation. In contrast, the FA-OM group showed a marginally significant upward trend during the first FA meditation phase. This would suggest that FA meditation rather than OM meditation may be associated with this skill. The score of *Being in The Moment* reflects the degree to which individuals can concentrate on what they do without falling into mind wandering or rumination. FA meditation training decreases the activation of the default mode network, which is associated with mind wandering [35]. As the FA meditation used in this study requires continuously focusing attention on one’s breath, it is reasonable that the training slightly fosters the skill of focusing on present experiences.

However, we also found that the score of this subscale increased at time 6 compared to the baseline in the OM-FA group, which was earlier than that in the FA-OM group (time 9). It is possible to consider that OM meditation implicitly cultivates the skill of being in the moment, which becomes apparent when combined with focused attention skills. Directing attention towards internal and external objects is a key characteristic of OM meditation. Remaining aware of what happened in the mind and the mind wandering toward things that are not now and here would be essential for staying in the moment. In addition, OM meditation would bolster the skill of stepping away from a stream of thought. OM meditation has been reported to gradually decrease emotional responses to stimuli by facilitating exposure [16]. Novices training in OM meditation at first would be likely to confront various sensations, feelings, and thoughts, including emotional responses. Individuals would not respond excessively to what they think or the wandering mind itself by practicing to direct their attention intentionally. This would prevent novices from becoming preoccupied with spontaneous thoughts and evaluations. Therefore, when practicing FA meditation after OM meditation, they could efficiently cultivate the skill to focus on present experiences and redirect attention when distracted by mind wandering or rumination.

### The effects of the order of meditation practice on mindfulness

In the basic principle of Buddhist meditation, the non-judgmental observation that brings about the notion of impermanence is based on deep concentration on a single object [18]. Therefore, it is recommended that one begin practicing OM meditation after sufficiently practicing FA meditation. Our results indicated that FA meditation within four weeks may increase trait mindfulness in novices more efficiently than OM meditation, and that practicing FA meditation prior to OM meditation enabled novices to develop mindfulness skills more quickly. This supports the validity of the traditional Buddhist discipline in the context of cultivating mindfulness. Zhang et al. [22] reported that trait mindfulness and mood improved in 4-week FA meditation period and were maintained during the following 4-week OM meditation period. As the target of attention and the goal of meditation are clearly defined in FA meditation, it is easier for novices to practice than OM meditation. Thus, novices could smoothly acquire mindfulness skills (i.e., acceptance and describing) through FA meditation, and then easily engage in OM meditation training. However, the order of the two meditation training sessions did not affect the level of most mindfulness skills at the end of the program, contrary to our hypothesis. In addition, we found that some skills might develop more rapidly through meditation training in the reverse order of discipline. In the case of a mindfulness program for a standard period (around eight weeks), order might not be a decisive factor on effectiveness.

We should also mention that it is difficult to separate the effects of these two types of meditation completely. Considering that FA and OM meditation share common components such as executive function [5,20], there may be nonspecific meditation effects. The *Tau-U* analysis in the present study showed that describing, acceptance, observing, and awareness skills increased from the first to the second phase at a medium effect size, regardless of the order of meditation practice. The amount of time spent on mindfulness meditation predicts daily mindful responding [36]. Our previous study also suggested that novices could engage in the second meditation more easily than the first meditation, even if the type of meditation was different [37]. Therefore, not only the order of meditation, but also the total period of meditation training influences the degree of mindfulness acquisition.

In conclusion, we found that the order of FA and OM meditation in mindfulness training may affect the process and degree of mindfulness skill acquisition in novices. To our knowledge, this is the first empirical study examining the effects of the order of FA and OM meditation training on mindfulness. The focused attention component is generally practiced before the open monitoring component in mindfulness training [22,38]. Although the order of the two meditations did not cause clear differences in the level of mindfulness score in most elements, it might influence the extent to which novices find meditation practices difficult.

### Limitations and future directions

The present study has several limitations. First, although the FA and OM meditations used incorporate core elements of these types of meditations, the procedures were not rigorously standardized. Thus, the consistency of the findings should be examined using a more standardized meditation procedure. In addition, because the study did not include 8-week FA or OM meditation conditions, we could not compare the individual and multiplier effects of the two meditations. The 8-week FA meditation, OM meditation, and mindfulness-based cognitive therapy (MBCT) have differential effects on cognitive and emotional regulation [1,10]. Thus, it is possible that the effects of FA and OM meditation on the acquisition of mindfulness interact regardless of the order of practice. Second, only one questionnaire was used as the index of trait mindfulness. Using objective indices, such as event‐related potential (ERP) [16] or behavioral measures of mind wandering [39] and mindfulness skills (e.g., Breath-Counting Task [40] and Mindful Awareness Task [41]), may enable the detection of more specific meditation effects. Third, as this study was a non-randomized controlled trial, some confounding factors may influence the effects of the intervention. For example, participants in the control group reported higher mindfulness skill than other groups at baseline. Differences of some cognitive skills or personality may exist between groups and affect the efficacy of each meditation training, as suggested in a previous study [21]. There may also be differences in some demographic variables between groups. Additionally, the repetitive measurement of the self-reported questionnaire could distort the results. Thus, it is necessary to compare the two types of meditation training considering such potential confounding factors using a randomized control design. Finally, because the sample size was small and limited to Japanese undergraduate and graduate students, caution is required when generalizing and considering the robustness of the findings. For example, the effects of FA or OM meditation training on some mindfulness skills in novices remained obscure as they changed at a marginally significant level. Future studies should examine whether these findings can be replicated in a larger sample, including a non-Japanese population.

## Supporting information

S1 Table The mean scores and standardized deviations of SFMS at each time point. (DOCX)

S1 Appendix Meditation instruction. (DOCX)

S1 File Anonymized participant information and SFMS scores. (DOCX)
