# Supplementary material for: The individual and sequential effect of focused attention and open monitoring meditation on mindfulness skills
Source: PLoS One. 2025 May 7;20(5):e0322537. doi: 10.1371/journal.pone.0322537 (PMC12057964; doi:10.1371/journal.pone.0322537)
Supplement: S1 Table — (DOCX) [file pone.0322537.s001.docx]

**S1 Table. The mean scores and standardized deviations of SFMS at each time point**

|  |  | time 1 | time 2 | time 3 | time 4 | time 5 | time 6 | time 7 | time 8 | time 9 |
| --- | --- | --- | --- | --- | --- | --- | --- | --- | --- | --- |
| Nonduality | FA-OM | 3.33 (0.92) | 3.37 (0.73) | 3.30 (0.66) | 3.44 (0.63) | 3.41 (0.44) | 3.41 (0.62) | 3.56 (0.70) | 3.63 (0.74) | 3.70 (0.73) |
|  | OM-FA | 3.24 (0.60) | 3.41 (0.49) | 3.41 (0.56) | 3.37 (0.55) | 3.44 (0.52) | 3.59 (0.49) | 3.44 (0.35) | 3.56 (0.38) | 3.67 (0.52) |
|  | Control | 3.11 (0.59) | 3.22 (0.80) | 3.30 (0.73) | 3.22 (0.72) | 3.22 (0.68) | 3.37 (0.88) | 3.30 (0.74) | 3.52 (0.77) | 3.44 (0.85) |
| Describing | FA-OM | 2.63 (0.82) | 2.81 (0.47) | 3.07 (0.68) | 3.44 (0.31) | 3.59 (0.47) | 3.44 (0.44) | 3.81 (0.45) | 3.89 (0.68) | 4.04 (0.33) |
|  | OM-FA | 2.81 (0.85) | 2.78 (0.65) | 2.93 (0.87) | 3.07 (0.81) | 3.15 (0.88) | 3.11 (0.86) | 3.44 (0.63) | 3.30 (0.84) | 3.56 (0.70) |
|  | Control | 3.93 (0.90) | 3.70 (0.60) | 3.59 (0.98) | 3.56 (0.72) | 3.44 (0.83) | 3.44 (0.85) | 3.56 (0.70) | 3.56 (0.86) | 3.78 (0.86) |
| Acceptance and  Nonreactivity | FA-OM | 2.72 (0.54) | 2.72 (0.54) | 2.80 (0.62) | 3.22 (0.48) | 3.37 (0.46) | 3.12 (0.50) | 3.30 (0.35) | 3.54 (0.62) | 3.33 (0.40) |
|  | OM-FA | 2.67 (0.68) | 2.90 (0.58) | 2.83 (0.89) | 2.90 (0.71) | 3.07 (0.72) | 3.17 (0.70) | 3.25 (0.74) | 3.07 (0.80) | 3.30 (0.77) |
|  | Control | 3.00 (0.79) | 2.86 (0.84) | 2.99 (0.98) | 2.80 (0.86) | 2.84 (0.83) | 2.77 (0.84) | 2.68 (0.81) | 2.83 (1.01) | 2.77 (0.74) |
| Objective  Observing | FA-OM | 3.11 (0.79) | 3.07 (0.70) | 2.74 (0.68) | 3.41 (0.54) | 3.67 (0.31) | 3.48 (0.42) | 3.67 (0.35) | 4.00 (0.50) | 3.81 (0.32) |
|  | OM-FA | 3.19 (0.50) | 3.30 (0.51) | 3.04 (0.76) | 3.15 (0.77) | 3.22 (0.79) | 3.30 (0.78) | 3.44 (0.57) | 3.59 (1.03) | 3.74 (0.84) |
|  | Control | 3.78 (0.85) | 3.74 (0.87) | 3.70 (0.76) | 3.59 (0.84) | 3.37 (0.79) | 3.33 (0.90) | 3.44 (0.90) | 3.56 (0.93) | 3.67 (0.75) |
| Awareness | FA-OM | 2.79 (0.50) | 2.98 (0.49) | 2.98 (0.53) | 3.30 (0.41) | 3.38 (0.45) | 3.46 (0.39) | 3.46 (0.39) | 3.62 (0.52) | 3.60 (0.39) |
|  | OM-FA | 2.78 (0.54) | 2.92 (0.60) | 2.97 (0.80) | 2.81 (0.71) | 3.00 (0.65) | 3.19 (0.75) | 3.33 (0.68) | 3.32 (0.53) | 3.41 (0.57) |
|  | Control | 3.30 (0.59) | 3.30 (0.73) | 3.21 (0.66) | 3.10 (0.66) | 3.05 (0.62) | 3.00 (0.59) | 3.19 (0.58) | 3.29 (0.64) | 3.22 (0.68) |
| Being  in the Moment | FA-OM | 2.83 (0.56) | 2.89 (0.53) | 2.80 (0.68) | 3.34 (0.58) | 3.37 (0.54) | 3.31 (0.54) | 3.30 (0.55) | 3.43 (0.72) | 3.46 (0.53) |
|  | OM-FA | 2.57 (0.62) | 2.96 (0.56) | 2.57 (0.70) | 2.78 (0.82) | 2.94 (0.66) | 3.28 (0.84) | 3.17 (0.82) | 3.11 (0.76) | 3.19 (0.80) |
|  | Control | 3.15 (0.76) | 3.22 (0.79) | 3.20 (0.89) | 2.91 (0.83) | 2.85 (0.92) | 3.07 (1.07) | 2.93 (1.00) | 3.11 (0.98) | 3.09 (0.79) |

The score of each subscale was divided by the number of items.

**S1 Appendix. Meditation Instruction**

FA Meditation:

Just allowing yourself to settle into the pelvis, allowing your spine to maintain a spontaneous S-shape curve, not stiff, and allowing your hands to rest gently in your lap by placing the palms down. Allowing your gaze to turn downwards, not moving restlessly.

Just taking a breath out slowly. At first, take about six breaths per minute. You may be aware of taking four times or less breaths per minute as you get used to it. There is no need to control your breaths, just keep breathing slowly. Starting to count your breath out silently from one to ten, then back to one again. Just keeping counting your breaths.

You may lose track of how many you have counted. Then, just starting to count from one again. Various thoughts may arise while you are counting your breaths. If you notice that your mind is wandering, just return your awareness back to the breath and counting it. You can start to count from the number you remember, or you can also start from one. Just repeating this process again and again. This method is called “Susoku-kan.” The breath and mind are tied together. Breathing fast makes it difficult to concentrate your mind. Just taking a breath out slowly.

OM Meditation:

Just allowing yourself to settle into the pelvis, allowing your spine to keep a spontaneous S-shape curve, not stiff, and allowing your hands to rest gently in your lap by placing the palms down. Allowing your gaze to turn downwards, not moving restlessly.

Just taking a breath in naturally while saying to yourself “inhaling,” observing the change of the physical sensations as if you are doing a live report yourself. For example, “Cold air is entering through the nose, passing through the nostrils…the throat…, filling the lungs.” Then, just taking a breath out while saying “exhaling,” observing the sensations of the body. For example, “Air is leaving the lungs through the throat…, slightly warmer breath is exiting through the nostrils.”

If you notice some physical sensations such as pain or numbness arise, just observing them too as if you are doing a live report yourself. For example, “There is a tingling sensation inside of the arm” or “There is a feeling of numbness and being bulging in the sole of the feet.” Similarly, just observing what arises in your mind such as thoughts, sleepiness, or irritation. For example, “I am thinking about something”, “I'm light-headed because of the sleepiness. My eyes are about to close”, or “The thought that I hope it to end soon is arising. There is fidgeting in the stomach area. The thought “it is not time to finish meditation yet” is arising, and there is a feeling of wanting to move the toes. My toes are moving. There are sounds of voices talking outside the room.” Just observing or doing a live report yourself of what it is now and here, as it is. Just conducting a report on whatever comes into the spotlight in your consciousness or attention. Just observing thoughts, physical sensations, feelings, and emotions as if you were doing a live report yourself.

**S1 File.**

| Participant | Group | Age | Gender | Past Experience | Time | Nonduality | Describing | Acceptance | Observing | Awareness | Moment |
| --- | --- | --- | --- | --- | --- | --- | --- | --- | --- | --- | --- |
| 1 | FA-OM | 18 | F | No | 1 | 2.333333 | 3.666667 | 3 | 2.333333 | 3.714286 | 3.833333 |
| 1 | FA-OM | 18 | F | No | 2 | 3 | 3 | 2.777778 | 2.333333 | 3.428571 | 3.666667 |
| 1 | FA-OM | 18 | F | No | 3 | 3 | 4 | 3 | 3 | 3.571429 | 3.666667 |
| 1 | FA-OM | 18 | F | No | 4 | 3 | 4 | 3.444444 | 3.333333 | 3 | 3.833333 |
| 1 | FA-OM | 18 | F | No | 5 | 3 | 4 | 3.444444 | 3.333333 | 3.857143 | 4 |
| 1 | FA-OM | 18 | F | No | 6 | 3 | 3 | 3.555556 | 3.666667 | 3.714286 | 4.333333 |
| 1 | FA-OM | 18 | F | No | 7 | 3 | 4 | 3.555556 | 3.666667 | 3.857143 | 3.833333 |
| 1 | FA-OM | 18 | F | No | 8 | 3 | 5 | 4.222222 | 4.666667 | 3.857143 | 4.666667 |
| 1 | FA-OM | 18 | F | No | 9 | 3 | 4 | 3.555556 | 4 | 3.571429 | 4 |
| 2 | FA-OM | 21 | M | No | 1 | 2 | 3.666667 | 3.444444 | 3.666667 | 3 | 2.5 |
| 2 | FA-OM | 21 | M | No | 2 | 2.333333 | 3.333333 | 3.444444 | 3.666667 | 2.714286 | 2.833333 |
| 2 | FA-OM | 21 | M | No | 3 | 2 | 3.666667 | 2.888889 | 2.333333 | 2.714286 | 2.333333 |
| 2 | FA-OM | 21 | M | No | 4 | 2 | 3.333333 | 3 | 3 | 2.714286 | 2.666667 |
| 2 | FA-OM | 21 | M | No | 5 | 2.666667 | 3 | 3.111111 | 3.333333 | 3 | 2.333333 |
| 2 | FA-OM | 21 | M | No | 6 | 2 | 3.666667 | 3 | 3.666667 | 3.428571 | 2.833333 |
| 2 | FA-OM | 21 | M | No | 7 | 2 | 4.666667 | 3.111111 | 3.666667 | 3.285714 | 2.333333 |
| 2 | FA-OM | 21 | M | No | 8 | 2 | 3.666667 | 3.333333 | 3.666667 | 3.571429 | 2.5 |
| 2 | FA-OM | 21 | M | No | 9 | 2 | 4 | 3.555556 | 4 | 3.571429 | 2.666667 |
| 3 | FA-OM | 20 | F | No | 1 | 4.333333 | 2 | 2 | 2.666667 | 2.142857 | 2 |
| 3 | FA-OM | 20 | F | No | 2 | 3 | 2.333333 | 2.444444 | 3 | 2.285714 | 2.5 |
| 3 | FA-OM | 20 | F | No | 3 | 3.333333 | 2.333333 | 2.111111 | 2.666667 | 3.142857 | 2.5 |
| 3 | FA-OM | 20 | F | No | 4 | 3.666667 | 3.333333 | 2.666667 | 4 | 3.714286 | 3.6 |
| 3 | FA-OM | 20 | F | No | 5 | 3.666667 | 3 | 2.555556 | 3.666667 | 3.714286 | 3.333333 |
| 3 | FA-OM | 20 | F | No | 6 | 3.666667 | 3.333333 | 3.111111 | 4 | 3.428571 | 3.666667 |
| 3 | FA-OM | 20 | F | No | 7 | 3 | 3.666667 | 2.777778 | 3.333333 | 3 | 2.833333 |
| 3 | FA-OM | 20 | F | No | 8 | 3.666667 | 4 | 3.222222 | 3.666667 | 3.428571 | 3.5 |
| 3 | FA-OM | 20 | F | No | 9 | 4.666667 | 4.666667 | 3 | 4.333333 | 4.142857 | 4 |
| 4 | FA-OM | 19 | M | No | 1 | 4 | 2.666667 | 2.555556 | 2 | 2.142857 | 3.166667 |
| 4 | FA-OM | 19 | M | No | 2 | 4.333333 | 3.333333 | 2.444444 | 2.333333 | 2.285714 | 3.333333 |
| 4 | FA-OM | 19 | M | No | 3 | 4 | 3.666667 | 2.333333 | 2 | 2.285714 | 3.166667 |
| 4 | FA-OM | 19 | M | No | 4 | 4 | 3.333333 | 2.555556 | 2.333333 | 3.285714 | 3.5 |
| 4 | FA-OM | 19 | M | No | 5 | 4 | 4 | 2.888889 | 4 | 3 | 3.666667 |
| 4 | FA-OM | 19 | M | No | 6 | 4 | 4 | 2.555556 | 3.333333 | 3.142857 | 3.666667 |
| 4 | FA-OM | 19 | M | No | 7 | 4.333333 | 4 | 3.222222 | 4 | 3.714286 | 4 |
| 4 | FA-OM | 19 | M | No | 8 | 4.333333 | 3.666667 | 3.555556 | 4 | 3.285714 | 3.666667 |
| 4 | FA-OM | 19 | M | No | 9 | 4 | 4 | 3.111111 | 3.666667 | 3.571429 | 3.666667 |
| 5 | FA-OM | 19 | F | No | 1 | 3.333333 | 2 | 2.111111 | 4 | 2.714286 | 3 |
| 5 | FA-OM | 19 | F | No | 2 | 3.666667 | 2 | 2.555556 | 4 | 3.571429 | 3 |
| 5 | FA-OM | 19 | F | No | 3 | 3.666667 | 3 | 3.777778 | 4 | 3.571429 | 3.833333 |
| 5 | FA-OM | 19 | F | No | 4 | 4 | 3.666667 | 4 | 4 | 4 | 4 |
| 5 | FA-OM | 19 | F | No | 5 | 3.333333 | 4 | 4 | 4 | 3.857143 | 4 |
| 5 | FA-OM | 19 | F | No | 6 | 3.666667 | 3 | 3.888889 | 4 | 4.142857 | 3.666667 |
| 5 | FA-OM | 19 | F | No | 7 | 3.666667 | 4 | 3.666667 | 4 | 4.142857 | 4 |
| 5 | FA-OM | 19 | F | No | 8 | 4.666667 | 5 | 4.555556 | 5 | 4.857143 | 4.5 |
| 5 | FA-OM | 19 | F | No | 9 | 3.666667 | 4 | 3.666667 | 4 | 4 | 3.666667 |
| 6 | FA-OM | 21 | F | No | 1 | 2.666667 | 2 | 2 | 2.333333 | 2.285714 | 2 |
| 6 | FA-OM | 21 | F | No | 2 | 2.333333 | 2.333333 | 2 | 2 | 2.571429 | 2 |
| 6 | FA-OM | 21 | F | No | 3 | 2.666667 | 2 | 2.111111 | 2 | 2.857143 | 2 |
| 6 | FA-OM | 21 | F | No | 4 | 3.666667 | 3.666667 | 3.555556 | 4 | 3.714286 | 3.666667 |
| 6 | FA-OM | 21 | F | No | 5 | 3.666667 | 4 | 3.888889 | 4 | 3.857143 | 3.666667 |
| 6 | FA-OM | 21 | F | No | 6 | 3 | 4 | 2.444444 | 3 | 3.857143 | 3 |
| 6 | FA-OM | 21 | F | No | 7 | 4 | 4 | 3.222222 | 4 | 3.571429 | 3.333333 |
| 6 | FA-OM | 21 | F | No | 8 | 3.666667 | 3.666667 | 3.111111 | 4 | 3.857143 | 3.333333 |
| 6 | FA-OM | 21 | F | No | 9 | 4 | 4 | 3.333333 | 3.333333 | 4 | 3.666667 |
| 7 | FA-OM | 19 | M | No | 1 | 3 | 2.666667 | 3.222222 | 3.666667 | 3 | 2.833333 |
| 7 | FA-OM | 19 | M | No | 2 | 4 | 3.333333 | 3.444444 | 3.333333 | 3.428571 | 3.333333 |
| 7 | FA-OM | 19 | M | No | 3 | 3.333333 | 3 | 3.555556 | 3.333333 | 3.285714 | 3.333333 |
| 7 | FA-OM | 19 | M | No | 4 | 3.666667 | 3 | 3.555556 | 3.333333 | 3.142857 | 3.833333 |
| 7 | FA-OM | 19 | M | No | 5 | 3.333333 | 3 | 3.333333 | 3.333333 | 3.142857 | 3.5 |
| 7 | FA-OM | 19 | M | No | 6 | 4 | 3 | 3.666667 | 3.333333 | 3.285714 | 3.333333 |
| 7 | FA-OM | 19 | M | No | 7 | 4 | 3 | 3.555556 | 3.333333 | 3 | 3 |
| 7 | FA-OM | 19 | M | No | 8 | 3.333333 | 3 | 3.666667 | 3.333333 | 3.142857 | 3 |
| 7 | FA-OM | 19 | M | No | 9 | 4 | 3.333333 | 3.555556 | 3.333333 | 2.857143 | 3.833333 |
| 8 | FA-OM | 21 | F | No | 1 | 3.333333 | 1.333333 | 2.777778 | 3 | 2.857143 | 3.166667 |
| 8 | FA-OM | 21 | F | No | 2 | 3.333333 | 2.666667 | 2 | 3 | 3.285714 | 2.166667 |
| 8 | FA-OM | 21 | F | No | 3 | 3.333333 | 2.333333 | 2.111111 | 2 | 2 | 1.833333 |
| 8 | FA-OM | 21 | F | No | 4 | 3 | 3 | 2.666667 | 3 | 2.857143 | 2.333333 |
| 8 | FA-OM | 21 | F | No | 5 | 3 | 3.333333 | 3.222222 | 3.333333 | 2.571429 | 2.666667 |
| 8 | FA-OM | 21 | F | No | 6 | 3.333333 | 3 | 2.555556 | 2.666667 | 2.714286 | 2.666667 |
| 8 | FA-OM | 21 | F | No | 7 | 4 | 3.333333 | 2.777778 | 3 | 3 | 2.833333 |
| 8 | FA-OM | 21 | F | No | 8 | 4 | 3 | 2.333333 | 3.666667 | 3 | 2.5 |
| 8 | FA-OM | 21 | F | No | 9 | 4 | 4 | 2.444444 | 3.666667 | 3.142857 | 2.5 |
| 9 | FA-OM | 18 | M | No | 1 | 5 | 3.666667 | 3.333333 | 4.333333 | 3.285714 | 3 |
| 9 | FA-OM | 18 | M | No | 2 | 4.333333 | 3 | 3.333333 | 4 | 3.285714 | 3.166667 |
| 9 | FA-OM | 18 | M | No | 3 | 4.333333 | 3.666667 | 3.333333 | 3.333333 | 3.428571 | 2.5 |
| 9 | FA-OM | 18 | M | No | 4 | 4 | 3.666667 | 3.555556 | 3.666667 | 3.285714 | 2.666667 |
| 9 | FA-OM | 18 | M | No | 5 | 4 | 4 | 3.888889 | 4 | 3.428571 | 3.166667 |
| 9 | FA-OM | 18 | M | No | 6 | 4 | 4 | 3.333333 | 3.666667 | 3.428571 | 2.666667 |
| 9 | FA-OM | 18 | M | No | 7 | 4 | 3.666667 | 3.777778 | 4 | 3.571429 | 3.5 |
| 9 | FA-OM | 18 | M | No | 8 | 4 | 4 | 3.888889 | 4 | 3.571429 | 3.166667 |
| 9 | FA-OM | 18 | M | No | 9 | 4 | 4.333333 | 3.777778 | 4 | 3.571429 | 3.166667 |
| 10 | OM-FA | 19 | F | No | 1 | 2.333333 | 2.333333 | 3.222222 | 3 | 3.428571 | 2.333333 |
| 10 | OM-FA | 19 | F | No | 2 | 2.666667 | 3 | 3.333333 | 3.666667 | 3.285714 | 2.5 |
| 10 | OM-FA | 19 | F | No | 3 | 2.666667 | 3.333333 | 3.666667 | 3.333333 | 3.428571 | 2.333333 |
| 10 | OM-FA | 19 | F | No | 4 | 3.333333 | 2.666667 | 3.777778 | 3.666667 | 3.285714 | 2.5 |
| 10 | OM-FA | 19 | F | No | 5 | 3.666667 | 3.333333 | 3.555556 | 3.666667 | 3.428571 | 2.5 |
| 10 | OM-FA | 19 | F | No | 6 | 3.333333 | 3 | 3.555556 | 3.666667 | 3.285714 | 2.666667 |
| 10 | OM-FA | 19 | F | No | 7 | 3 | 3.333333 | 3.555556 | 3.666667 | 3.285714 | 2.666667 |
| 10 | OM-FA | 19 | F | No | 8 | 3.333333 | 3.333333 | 3.666667 | 3.666667 | 3.571429 | 2.666667 |
| 10 | OM-FA | 19 | F | No | 9 | 3.666667 | 3.333333 | 3.555556 | 3.666667 | 3.428571 | 2.666667 |
| 11 | OM-FA | 20 | F | Yes (Meditation) | 1 | 3.333333 | 2.333333 | 1.666667 | 2.666667 | 2.142857 | 1.5 |
| 11 | OM-FA | 20 | F | Yes (Meditation) | 2 | 3.666667 | 3.333333 | 2.888889 | 2.666667 | 2.857143 | 3.333333 |
| 11 | OM-FA | 20 | F | Yes (Meditation) | 3 | 4 | 3.666667 | 1.666667 | 2.666667 | 3.571429 | 1.5 |
| 11 | OM-FA | 20 | F | Yes (Meditation) | 4 | 2.666667 | 3.666667 | 2.444444 | 2.666667 | 3 | 1 |
| 11 | OM-FA | 20 | F | Yes (Meditation) | 5 | 2.666667 | 3.666667 | 2.777778 | 2.333333 | 2.714286 | 1.666667 |
| 11 | OM-FA | 20 | F | Yes (Meditation) | 6 | 3 | 2.333333 | 2.222222 | 2 | 2.571429 | 1.333333 |
| 11 | OM-FA | 20 | F | Yes (Meditation) | 7 | 3.333333 | 3.666667 | 2.333333 | 3 | 3.142857 | 1.5 |
| 11 | OM-FA | 20 | F | Yes (Meditation) | 8 | 3 | 3.666667 | 2.777778 | 4.333333 | 3.285714 | 1.666667 |
| 11 | OM-FA | 20 | F | Yes (Meditation) | 9 | 3.333333 | 4 | 3.777778 | 4.666667 | 3.571429 | 1.666667 |
| 12 | OM-FA | 18 | M | No | 1 | 4 | 3 | 3.777778 | 3.666667 | 2.571429 | 3.666667 |
| 12 | OM-FA | 18 | M | No | 2 | 4 | 2 | 3.555556 | 3.666667 | 2.571429 | 4 |
| 12 | OM-FA | 18 | M | No | 3 | 4 | 2.333333 | 3.777778 | 4 | 2.571429 | 4 |
| 12 | OM-FA | 18 | M | No | 4 | 4 | 2.333333 | 3.222222 | 3.666667 | 2 | 3.833333 |
| 12 | OM-FA | 18 | M | No | 5 | 4 | 2 | 3.666667 | 3.666667 | 2.428571 | 3.833333 |
| 12 | OM-FA | 18 | M | No | 6 | 4.333333 | 3 | 3.888889 | 4 | 3.142857 | 4 |
| 12 | OM-FA | 18 | M | No | 7 | 4 | 3.333333 | 4.111111 | 4 | 3.428571 | 4 |
| 12 | OM-FA | 18 | M | No | 8 | 4 | 3.666667 | 4.111111 | 4.333333 | 3.571429 | 4.333333 |
| 12 | OM-FA | 18 | M | No | 9 | 4.666667 | 3.666667 | 4 | 4.666667 | 3.714286 | 4.5 |
| 13 | OM-FA | 19 | F | No | 1 | 2.5 | 2 | 2.777778 | 2.333333 | 2.285714 | 3.166667 |
| 13 | OM-FA | 19 | F | No | 2 | 3.333333 | 2 | 2.888889 | 3.333333 | 2.714286 | 3 |
| 13 | OM-FA | 19 | F | No | 3 | 3 | 2 | 2.888889 | 3 | 3.428571 | 2.5 |
| 13 | OM-FA | 19 | F | No | 4 | 3 | 3 | 2.666667 | 3 | 3 | 2.833333 |
| 13 | OM-FA | 19 | F | No | 5 | 3.333333 | 3 | 3.111111 | 3 | 3.142857 | 2.833333 |
| 13 | OM-FA | 19 | F | No | 6 | 4 | 3.666667 | 3.555556 | 3.666667 | 3.714286 | 4 |
| 13 | OM-FA | 19 | F | No | 7 | 3.666667 | 3.666667 | 3.111111 | 3.666667 | 3.428571 | 3.333333 |
| 13 | OM-FA | 19 | F | No | 8 | 3.666667 | 3 | 3.333333 | 3.666667 | 3.714286 | 3.5 |
| 13 | OM-FA | 19 | F | No | 9 | 4 | 3.666667 | 3.888889 | 4 | 3.714286 | 4 |
| 14 | OM-FA | 19 | M | No | 1 | 4 | 3.333333 | 3 | 3.666667 | 3.142857 | 2.666667 |
| 14 | OM-FA | 19 | M | No | 2 | 4 | 2.666667 | 3.888889 | 4 | 3.285714 | 3.5 |
| 14 | OM-FA | 19 | M | No | 3 | 4 | 3.333333 | 3.888889 | 3.333333 | 3.285714 | 3.5 |
| 14 | OM-FA | 19 | M | No | 4 | 4 | 2.666667 | 4 | 3.666667 | 3 | 3.833333 |
| 14 | OM-FA | 19 | M | No | 5 | 4 | 2.666667 | 4 | 4 | 3.285714 | 4 |
| 14 | OM-FA | 19 | M | No | 6 | 4 | 3.333333 | 4 | 3.666667 | 3.285714 | 4 |
| 14 | OM-FA | 19 | M | No | 7 | 3.666667 | 3 | 4 | 3.666667 | 3.285714 | 3.833333 |
| 14 | OM-FA | 19 | M | No | 8 | 4 | 2.666667 | 4.111111 | 4 | 3.285714 | 3.666667 |
| 14 | OM-FA | 19 | M | No | 9 | 4 | 3 | 4.111111 | 4 | 3.142857 | 3.666667 |
| 15 | OM-FA | 19 | F | No | 1 | 3 | 2 | 2.555556 | 2.666667 | 2.428571 | 3 |
| 15 | OM-FA | 19 | F | No | 2 | 3 | 2.333333 | 2.333333 | 2.333333 | 2.285714 | 3 |
| 15 | OM-FA | 19 | F | No | 3 | 3.333333 | 1.666667 | 1.888889 | 1.666667 | 1.714286 | 2.5 |
| 15 | OM-FA | 19 | F | No | 4 | 3 | 2.666667 | 2.111111 | 1.666667 | 1.857143 | 2.666667 |
| 15 | OM-FA | 19 | F | No | 5 | 3.666667 | 2.333333 | 2 | 2 | 2.428571 | 2.833333 |
| 15 | OM-FA | 19 | F | No | 6 | 3.666667 | 2 | 2.444444 | 2 | 2.285714 | 3.166667 |
| 15 | OM-FA | 19 | F | No | 7 | 3.666667 | 2.333333 | 2.555556 | 2.333333 | 2.571429 | 3.166667 |
| 15 | OM-FA | 19 | F | No | 8 | 3.666667 | 2 | 1.666667 | 1 | 2.428571 | 2.333333 |
| 15 | OM-FA | 19 | F | No | 9 | 3 | 2.666667 | 1.777778 | 2 | 2.142857 | 3 |
| 16 | OM-FA | 22 | F | Yes (Yoga) | 1 | 4 | 4 | 2.888889 | 3.666667 | 3.857143 | 2 |
| 16 | OM-FA | 22 | F | Yes (Yoga) | 2 | 4 | 3.333333 | 2.888889 | 3.666667 | 4.142857 | 2 |
| 16 | OM-FA | 22 | F | Yes (Yoga) | 3 | 4 | 4 | 3.666667 | 4 | 4.285714 | 2.166667 |
| 16 | OM-FA | 22 | F | Yes (Yoga) | 4 | 4.333333 | 4.666667 | 3.444444 | 4.333333 | 4.285714 | 2.666667 |
| 16 | OM-FA | 22 | F | Yes (Yoga) | 5 | 4 | 4.666667 | 3.888889 | 4.333333 | 4.285714 | 3.166667 |
| 16 | OM-FA | 22 | F | Yes (Yoga) | 6 | 4 | 4.666667 | 3.888889 | 4.333333 | 4.857143 | 3.833333 |
| 16 | OM-FA | 22 | F | Yes (Yoga) | 7 | 3.666667 | 4.666667 | 4.333333 | 4.333333 | 4.857143 | 4.166667 |
| 16 | OM-FA | 22 | F | Yes (Yoga) | 8 | 4 | 4.666667 | 3 | 4.666667 | 4.142857 | 3.5 |
| 16 | OM-FA | 22 | F | Yes (Yoga) | 9 | 4 | 5 | 3.333333 | 4.333333 | 4.285714 | 3.166667 |
| 17 | OM-FA | 19 | M | No | 1 | 3 | 2 | 2.666667 | 3.333333 | 2.428571 | 2.666667 |
| 17 | OM-FA | 19 | M | No | 2 | 3 | 2.333333 | 2.222222 | 3.333333 | 2 | 2.666667 |
| 17 | OM-FA | 19 | M | No | 3 | 3 | 2 | 2.222222 | 2 | 1.857143 | 2.5 |
| 17 | OM-FA | 19 | M | No | 4 | 3 | 2 | 2.666667 | 2.333333 | 2.142857 | 3.333333 |
| 17 | OM-FA | 19 | M | No | 5 | 3 | 2.333333 | 2.666667 | 2.333333 | 2 | 3 |
| 17 | OM-FA | 19 | M | No | 6 | 3 | 2 | 2.555556 | 3 | 2.285714 | 3.666667 |
| 17 | OM-FA | 19 | M | No | 7 | 3 | 3 | 2.888889 | 3 | 2.285714 | 3.5 |
| 17 | OM-FA | 19 | M | No | 8 | 3.333333 | 2.333333 | 3 | 3 | 2.428571 | 3.5 |
| 17 | OM-FA | 19 | M | No | 9 | 3.333333 | 2.666667 | 3 | 2.666667 | 3 | 3.5 |
| 18 | OM-FA | 18 | F | No | 1 | 3 | 4.333333 | 1.444444 | 3.666667 | 2.714286 | 2.166667 |
| 18 | OM-FA | 18 | F | No | 2 | 3 | 4 | 2.111111 | 3 | 3.142857 | 2.666667 |
| 18 | OM-FA | 18 | F | No | 3 | 2.666667 | 4 | 1.777778 | 3.333333 | 2.571429 | 2.166667 |
| 18 | OM-FA | 18 | F | No | 4 | 3 | 4 | 1.777778 | 3.333333 | 2.714286 | 2.333333 |
| 18 | OM-FA | 18 | F | No | 5 | 2.666667 | 4.333333 | 2 | 3.666667 | 3.285714 | 2.666667 |
| 18 | OM-FA | 18 | F | No | 6 | 3 | 4 | 2.444444 | 3.333333 | 3.285714 | 2.833333 |
| 18 | OM-FA | 18 | F | No | 7 | 3 | 4 | 2.333333 | 3.333333 | 3.714286 | 2.333333 |
| 18 | OM-FA | 18 | F | No | 8 | 3 | 4.333333 | 2 | 3.666667 | 3.428571 | 2.833333 |
| 18 | OM-FA | 18 | F | No | 9 | 3 | 4 | 2.222222 | 3.666667 | 3.714286 | 2.5 |
| 19 | Control | 23 | F | No | 1 | 4 | 5 | 3.888889 | 5 | 4.428571 | 3.833333 |
| 19 | Control | 23 | F | No | 2 | 4 | 4.666667 | 4 | 5 | 4.142857 | 4 |
| 19 | Control | 23 | F | No | 3 | 4 | 5 | 4.222222 | 5 | 4 | 3.833333 |
| 19 | Control | 23 | F | No | 4 | 4 | 4.666667 | 4.111111 | 4.666667 | 4.285714 | 3.333333 |
| 19 | Control | 23 | F | No | 5 | 4 | 5 | 3 | 3.666667 | 3.571429 | 2.333333 |
| 19 | Control | 23 | F | No | 6 | 4.333333 | 5 | 3.666667 | 5 | 4 | 3.333333 |
| 19 | Control | 23 | F | No | 7 | 4.333333 | 5 | 3.111111 | 4.666667 | 4.285714 | 2.833333 |
| 19 | Control | 23 | F | No | 8 | 4.333333 | 5 | 4.333333 | 5 | 4.428571 | 4.166667 |
| 19 | Control | 23 | F | No | 9 | 4.333333 | 5 | 3.333333 | 4.666667 | 4.428571 | 3.333333 |
| 20 | Control | 23 | F | No | 1 | 2.666667 | 3.666667 | 2 | 2 | 2.142857 | 3 |
| 20 | Control | 23 | F | No | 2 | 3 | 3.666667 | 1.888889 | 2 | 2 | 2.333333 |
| 20 | Control | 23 | F | No | 3 | 3 | 2.666667 | 2.111111 | 2.333333 | 2.285714 | 3 |
| 20 | Control | 23 | F | No | 4 | 3 | 2.666667 | 2 | 2 | 2.285714 | 2.5 |
| 20 | Control | 23 | F | No | 5 | 3 | 2.666667 | 2 | 2 | 2 | 2.5 |
| 20 | Control | 23 | F | No | 6 | 3 | 2.666667 | 2 | 2 | 2.285714 | 2.5 |
| 20 | Control | 23 | F | No | 7 | 3 | 2.666667 | 2 | 2 | 2.285714 | 2.666667 |
| 20 | Control | 23 | F | No | 8 | 3 | 2.666667 | 2 | 2 | 2.285714 | 2.666667 |
| 20 | Control | 23 | F | No | 9 | 3 | 2.666667 | 2 | 2 | 2 | 2.5 |
| 21 | Control | 20 | F | No | 1 | 3.666667 | 2.666667 | 3.888889 | 4 | 3.428571 | 4 |
| 21 | Control | 20 | F | No | 2 | 4 | 2.666667 | 3.666667 | 3.333333 | 2.857143 | 3.666667 |
| 21 | Control | 20 | F | No | 3 | 4 | 2 | 3.777778 | 4 | 2.857143 | 3.833333 |
| 21 | Control | 20 | F | No | 4 | 3.333333 | 2.666667 | 3.555556 | 4 | 2.714286 | 3.666667 |
| 21 | Control | 20 | F | No | 5 | 3.333333 | 2.666667 | 3.666667 | 4 | 2.857143 | 4 |
| 21 | Control | 20 | F | No | 6 | 3 | 2.666667 | 3.777778 | 3.333333 | 2.714286 | 4 |
| 21 | Control | 20 | F | No | 7 | 3.666667 | 3 | 3.666667 | 4 | 2.857143 | 4 |
| 21 | Control | 20 | F | No | 8 | 3.333333 | 2.666667 | 3.666667 | 3.666667 | 2.714286 | 3.666667 |
| 21 | Control | 20 | F | No | 9 | 3 | 3 | 3.555556 | 3.666667 | 2.714286 | 4 |
| 22 | Control | 20 | F | No | 1 | 2.666667 | 5 | 2.888889 | 4.333333 | 3.285714 | 3.833333 |
| 22 | Control | 20 | F | No | 2 | 2.333333 | 4.333333 | 2.888889 | 4.333333 | 3.428571 | 3.5 |
| 22 | Control | 20 | F | No | 3 | 2.333333 | 4.666667 | 3 | 4.333333 | 3.142857 | 3.666667 |
| 22 | Control | 20 | F | No | 4 | 2.333333 | 4.333333 | 3.111111 | 4.333333 | 3.142857 | 3.5 |
| 22 | Control | 20 | F | No | 5 | 2 | 4.333333 | 3.222222 | 4.333333 | 3 | 3.666667 |
| 22 | Control | 20 | F | No | 6 | 2 | 4.333333 | 2.888889 | 4 | 3.142857 | 3.833333 |
| 22 | Control | 20 | F | No | 7 | 2.333333 | 4 | 2.888889 | 4 | 3.142857 | 3.833333 |
| 22 | Control | 20 | F | No | 8 | 2.333333 | 4 | 3.111111 | 4 | 3.142857 | 4.166667 |
| 22 | Control | 20 | F | No | 9 | 2 | 4.333333 | 2.888889 | 4 | 3.285714 | 4 |
| 23 | Control | 19 | F | No | 1 | 3.333333 | 5 | 4.222222 | 4 | 3.285714 | 3.5 |
| 23 | Control | 19 | F | No | 2 | 4.333333 | 4 | 4.111111 | 4.666667 | 3.714286 | 4.5 |
| 23 | Control | 19 | F | No | 3 | 4.333333 | 4 | 4.444444 | 4.333333 | 3.714286 | 4.666667 |
| 23 | Control | 19 | F | No | 4 | 4 | 4 | 3.888889 | 4.333333 | 3.571429 | 4.166667 |
| 23 | Control | 19 | F | No | 5 | 4 | 3 | 4.555556 | 4 | 3.857143 | 4.333333 |
| 23 | Control | 19 | F | No | 6 | 4.666667 | 4 | 4.111111 | 4 | 3.428571 | 4.666667 |
| 23 | Control | 19 | F | No | 7 | 3.333333 | 4 | 4.222222 | 4.333333 | 3.857143 | 4.333333 |
| 23 | Control | 19 | F | No | 8 | 3.666667 | 3 | 4.222222 | 4.666667 | 3.571429 | 4.166667 |
| 23 | Control | 19 | F | No | 9 | 3 | 5 | 4 | 4.666667 | 3.857143 | 4 |
| 24 | Control | 19 | M | Yes (Meditation) | 1 | 2.333333 | 3.333333 | 2.111111 | 4 | 2.857143 | 2 |
| 24 | Control | 19 | M | Yes (Meditation) | 2 | 2.666667 | 3.333333 | 1.666667 | 3 | 3.285714 | 2.666667 |
| 24 | Control | 19 | M | Yes (Meditation) | 3 | 2.666667 | 3 | 1.333333 | 3.333333 | 3 | 1.833333 |
| 24 | Control | 19 | M | Yes (Meditation) | 4 | 4 | 3.666667 | 1.555556 | 4 | 2.428571 | 1.833333 |
| 24 | Control | 19 | M | Yes (Meditation) | 5 | 4 | 3.333333 | 1.888889 | 3.333333 | 2.857143 | 1.5 |
| 24 | Control | 19 | M | Yes (Meditation) | 6 | 4.333333 | 2.666667 | 1.666667 | 3.333333 | 2.714286 | 2 |
| 24 | Control | 19 | M | Yes (Meditation) | 7 | 4 | 3.333333 | 1.888889 | 4 | 2.857143 | 1.666667 |
| 24 | Control | 19 | M | Yes (Meditation) | 8 | 5 | 4.333333 | 1.222222 | 4 | 3.428571 | 1.833333 |
| 24 | Control | 19 | M | Yes (Meditation) | 9 | 5 | 3.333333 | 1.555556 | 3.666667 | 3.142857 | 1.833333 |
| 25 | Control | 18 | F | No | 1 | 2.333333 | 3.666667 | 2.444444 | 4 | 3.571429 | 2.5 |
| 25 | Control | 18 | F | No | 2 | 2.333333 | 4 | 2.666667 | 4 | 4.142857 | 2.666667 |
| 25 | Control | 18 | F | No | 3 | 2.666667 | 4 | 3.222222 | 3.666667 | 4 | 2.5 |
| 25 | Control | 18 | F | No | 4 | 2 | 3.333333 | 2.111111 | 3.333333 | 3.142857 | 1.833333 |
| 25 | Control | 18 | F | No | 5 | 2.333333 | 3 | 2.444444 | 3.666667 | 3.714286 | 2.166667 |
| 25 | Control | 18 | F | No | 6 | 2.333333 | 3 | 2.222222 | 2 | 3.285714 | 2.333333 |
| 25 | Control | 18 | F | No | 7 | 2 | 3.666667 | 1.777778 | 2.666667 | 3.285714 | 2.166667 |
| 25 | Control | 18 | F | No | 8 | 3 | 4 | 2.333333 | 3.333333 | 3.571429 | 2.166667 |
| 25 | Control | 18 | F | No | 9 | 3.666667 | 4 | 2.555556 | 3.666667 | 3.428571 | 3.166667 |
| 26 | Control | 22 | M | No | 1 | 3.333333 | 4.333333 | 3.222222 | 2.666667 | 3.714286 | 3.666667 |
| 26 | Control | 22 | M | No | 2 | 2.333333 | 3.666667 | 2.555556 | 3.333333 | 3.857143 | 3.666667 |
| 26 | Control | 22 | M | No | 3 | 2.666667 | 4.333333 | 2.555556 | 3.333333 | 3.714286 | 3.5 |
| 26 | Control | 22 | M | No | 4 | 2.666667 | 4 | 2.666667 | 3 | 3.857143 | 3.333333 |
| 26 | Control | 22 | M | No | 5 | 3 | 4.333333 | 2.777778 | 3.333333 | 3.428571 | 3.166667 |
| 26 | Control | 22 | M | No | 6 | 3 | 4 | 2.555556 | 3.333333 | 3.428571 | 3.833333 |
| 26 | Control | 22 | M | No | 7 | 3 | 3.666667 | 2.555556 | 3 | 3.428571 | 3.5 |
| 26 | Control | 22 | M | No | 8 | 3 | 4 | 2.444444 | 2.666667 | 3.857143 | 3.5 |
| 26 | Control | 22 | M | No | 9 | 3 | 4 | 2.777778 | 3.333333 | 3.571429 | 3 |
| 27 | Control | 19 | F | No | 1 | 3.666667 | 2.666667 | 2.333333 | 4 | 3 | 2 |
| 27 | Control | 19 | F | No | 2 | 4 | 3 | 2.333333 | 4 | 2.285714 | 2 |
| 27 | Control | 19 | F | No | 3 | 4 | 2.666667 | 2.222222 | 3 | 2.142857 | 2 |
| 27 | Control | 19 | F | No | 4 | 3.666667 | 2.666667 | 2.222222 | 2.666667 | 2.428571 | 2 |
| 27 | Control | 19 | F | No | 5 | 3.333333 | 2.666667 | 2 | 2 | 2.142857 | 2 |
| 27 | Control | 19 | F | No | 6 | 3.666667 | 2.666667 | 2 | 3 | 2 | 1.166667 |
| 27 | Control | 19 | F | No | 7 | 4 | 2.666667 | 2 | 2.333333 | 2.714286 | 1.333333 |
| 27 | Control | 19 | F | No | 8 | 4 | 2.333333 | 2.111111 | 2.666667 | 2.571429 | 1.666667 |
| 27 | Control | 19 | F | No | 9 | 4 | 2.666667 | 2.222222 | 3.333333 | 2.571429 | 2 |
